# Multidrug-Resistant Tuberculosis in Rural Eastern Cape, South Africa: Clinical, Bacteriological, and Programmatic Predictors of Poor Treatment Outcomes

**DOI:** 10.3390/microorganisms14030690

**Published:** 2026-03-18

**Authors:** Mbulelo Cebisa, Ntandazo Dlatu, Mojisola Clara Hosu, Teke Apalata, Lindiwe Modest Faye

**Affiliations:** 1 TB and Associated Research and Innovation Platform (TARIP), WSU-TB Research Group, School of Pathology, Faculty of Medicine and Health Sciences, Walter Sisulu University, Mthatha 5099, South Africa; 221279962@mywsu.ac.za (M.C.); mhosu@wsu.ac.za (M.C.H.); tapalata@wsu.ac.za (T.A.); 2 School of Public Health, Faculty of Medicine and Health Sciences, Walter Sisulu University, Mthatha 5099, South Africa; ndlatu@wsu.co.za

**Keywords:** multidrug-resistant tuberculosis, HIV coinfection, bacillary load, retreatment tuberculosis, culture positivity, clinical governance, drug-resistance predictors, treatment failure, rural health systems

## Abstract

Drug-resistant tuberculosis (DR-TB), particularly multidrug-resistant TB (MDR-TB), remains a significant public health challenge in rural South Africa, where diagnostic and treatment infrastructure is limited. This study aimed to assess resistance patterns, bacillary load, treatment outcomes, and predictors of MDR-TB in the O.R. Tambo District of the Eastern Cape Province. Although isoniazid monoresistant TB (Hr-TB) was identified, its analysis was descriptive due to the limited sample size. A retrospective cohort analysis was conducted on bacteriologically confirmed TB cases (n = 477) diagnosed between 2020 and 2022. The data collected included demographic and clinical variables, smear and culture results, resistance patterns, and treatment outcomes. Drug resistance was categorized as MDR-TB, Hr-TB, or fully susceptible. Outcomes were classified as favorable, unfavorable, lost to follow-up, or ongoing. Logistic regression identified predictors of MDR-TB. DR-TB prevalence was 11.3% (n = 54), with MDR-TB accounting for 10.7% (n = 51) and Hr-TB for 0.6% (n = 3). Prior TB treatment was significantly associated with MDR-TB (adjusted odds ratio [aOR] 4.45, 95% CI: 1.89–10.48). Smear positivity was associated with MDR-TB in univariate analysis (OR 5.0), although its effect diminished in multivariable analysis (aOR 0.40, 95% CI: 0.12–1.36), suggesting confounding by bacillary load. Culture positivity was a strong independent predictor (aOR 27.71, 95% CI: 8.84–86.85), indicating a higher mycobacterial burden among MDR-TB cases. MDR-TB patients had significantly poorer treatment outcomes, with a high rate of unfavorable outcomes and loss to follow-up. MDR-TB dominates the resistance landscape in this rural district, primarily driven by prior treatment and high bacillary loads. The study highlights the need for targeted interventions, including enhanced diagnostic capacity, improved clinical governance, and community-based support systems, to optimize the detection and management of MDR-TB.

## 1. Introduction

Tuberculosis (TB) remains one of the leading infectious causes of mortality worldwide and continues to pose a significant public health challenge despite decades of global control efforts. According to the World Health Organization (WHO), an estimated 10.6 million people developed TB in 2022, resulting in approximately 1.3 million deaths among HIV-negative individuals and an additional 167,000 deaths among people living with HIV. Drug-resistant tuberculosis (DR-TB), particularly multidrug-resistant TB (MDR-TB) and rifampicin-resistant TB (RR-TB), represent a major threat to global TB elimination efforts because of its complex treatment regimens, higher treatment costs, and poorer clinical outcomes compared with drug-susceptible disease. In 2022 alone, nearly 500,000 new cases of MDR/RR-TB were reported globally, reflecting a continued increase compared with previous years and highlighting persistent gaps in early detection and treatment access [1]. South Africa remains among the countries with the highest TB burden globally. It is one of the eight nations that collectively account for approximately two-thirds of all TB cases worldwide. The epidemic in South Africa is further compounded by a high prevalence of HIV infection, which significantly increases susceptibility to TB infection and progression to active disease. Within the country, the Eastern Cape Province consistently reports some of the highest TB incidence rates, particularly in rural districts where health system constraints and socioeconomic vulnerabilities contribute to ongoing transmission. High levels of poverty, overcrowded housing, limited healthcare access, and delayed diagnosis continue to fuel TB transmission in these communities. Moreover, the coexistence of TB and HIV creates additional clinical and programmatic challenges, including increased disease severity, diagnostic complexity, and higher rates of treatment failure [2]. Drug resistance further complicates TB control efforts by reducing treatment effectiveness and increasing the risk of prolonged infectiousness within communities. Patients with MDR-TB often require extended treatment regimens that are more toxic, expensive, and difficult to complete than standard first-line therapies. Consequently, DR-TB not only affects individual patient outcomes but also undermines community-based TB control strategies by facilitating ongoing transmission of resistant strains. In many high-burden settings, delays in detecting drug resistance allow patients to remain on ineffective treatment regimens, thereby increasing the risk of disease progression and community spread [3,4]. Although MDR-TB has received considerable programmatic attention over the past decade, increasing evidence suggests that isoniazid monoresistant TB (Hr-TB) represents an important yet underrecognized component of the drug-resistance spectrum. Isoniazid resistance often leads to treatment failure, relapse, and the eventual development of multidrug resistance when it is not properly identified and managed. Molecular resistance mechanisms primarily result from mutations in the *katG* and *inhA* genes, which confer distinct resistance phenotypes with significant effects on treatment plans, drug susceptibility, and clinical outcomes. For instance, *katG* mutations usually confer high-level resistance to isoniazid, while *inhA* mutations tend to confer low-level resistance, though they can also lead to cross-resistance to ethionamide. These molecular differences highlight the crucial need for accurate diagnostic testing and mutation-specific treatment approaches to enhance treatment success rates [5,6].

Despite the clinical significance of isoniazid resistance, routine surveillance systems in many high-burden countries primarily focus on detecting rifampicin resistance as a proxy for MDR-TB. While this approach has improved the rapid identification of multidrug-resistant cases, it may overlook patients with Hr-TB who remain susceptible to rifampicin but still require modified treatment regimens. Studies across sub-Saharan Africa have demonstrated that Hr-TB is frequently underdiagnosed due to limitations in diagnostic capacity, including restricted access to molecular testing and incomplete resistance profiling. As a result, many patients may receive suboptimal treatment regimens, which can contribute to treatment failure, ongoing transmission, and the amplification of resistance within communities [7,8,9].

The Eastern Cape Province presents a particularly complex environment for TB control. The region is characterized by constrained laboratory infrastructure, limited diagnostic resources, and a high burden of TB/HIV co-infection. Many healthcare facilities serve geographically dispersed populations, requiring the transport of specimens to centralized laboratories for culture and molecular testing. These logistical challenges may lead to diagnostic delays, resulting in higher bacillary loads at presentation and poorer treatment outcomes. Previous research from this region has documented elevated bacterial loads among patients with DR-TB as well as increased rates of unfavorable treatment outcomes among individuals with MDR-TB [10,11]. However, important knowledge gaps remain regarding the prevalence, molecular mutation profiles, and clinical implications of isoniazid monoresistance in rural programmatic cohorts. In addition to biological determinants of resistance, health systems and social factors also play a crucial role in shaping TB treatment outcomes. Clinical governance practices, such as adherence to diagnostic algorithms, routine monitoring of treatment adherence, and systematic auditing of retreatment cases, are essential to ensuring the effective implementation of TB control programs. Similarly, community engagement initiatives, including stigma reduction, patient education, and support provided by community health workers (CHWs), are increasingly recognized as key components of successful TB control strategies. In rural settings where healthcare access may be limited, CHWs often serve as an important bridge between healthcare facilities and communities, supporting treatment adherence and facilitating early identification of patients at risk of treatment interruption [12,13]. Given these challenges, there is an urgent need for context-specific evidence that combines clinical, bacteriological, and programmatic insights to better understand what drives DR-TB in rural South Africa. In this setting, we carried out a retrospective cohort study of patients diagnosed with DR-TB in the O.R. Tambo District of the Eastern Cape Province. The goals of this study were to identify the patterns and predictors of isoniazid monoresistance and MDR-TB, investigate their link to treatment outcomes, and analyze the molecular distribution of isoniazid resistance mutations. By combining analyses of clinical management practices with community engagement strategies, this study seeks to produce actionable evidence to improve programmatic responses, reduce retreatment-driven MDR-TB, and foster trust in TB care services within rural communities.

## 2. Methodology

### 2.1. Study Design and Setting

This study employed a retrospective cohort design to analyze bacteriologically confirmed TB cases diagnosed between 2020 and 2024 in the O.R. Tambo District of the Eastern Cape Province, South Africa. The district represents a predominantly rural setting with a high burden of TB and HIV co-infection. Healthcare services in the district are delivered through a network of primary healthcare clinics and district hospitals operating within the national TB control program. These facilities serve geographically dispersed communities, and diagnostic services for DR-TB are partly centralized at district laboratories. The rural nature of the district, combined with socioeconomic challenges and constrained health system resources, poses unique barriers to timely TB diagnosis and management.

### 2.2. Study Population

The study included 477 patient records of individuals diagnosed with bacteriologically confirmed TB during the study period. Records were obtained from routine clinical and laboratory databases maintained by healthcare facilities in the district. Inclusion criteria consisted of patients with complete laboratory data for smear microscopy, culture testing, and line probe assay (LPA) results. Records lacking key variables required for regression analysis were excluded from multivariable models but retained for descriptive analyses to preserve the overall representation of the study population.

### 2.3. Diagnostic Capacity and Testing Procedures

Diagnostic evaluation for TB at the participating facilities included smear microscopy performed at peripheral health facilities. In contrast, more advanced diagnostic testing, including culture and LPA for drug-resistance detection, was conducted at centralized district laboratories. Specimens collected at peripheral facilities were transported to these laboratories for further analysis. Due to the geographic dispersion of healthcare facilities and reliance on specimen transport systems, turnaround times for diagnostic results varied across facilities. These logistical factors may have contributed to delays in resistance confirmation and potentially influenced bacillary load at the time of diagnosis, as reflected in smear and culture positivity among patients.

### 2.4. Drug Resistance Classification

Drug resistance patterns were classified based on laboratory-confirmed results from LPA testing. Multidrug-resistant TB (MDR-TB) was defined as resistance to both isoniazid (INH) and rifampicin (RIF). Isoniazid monoresistant TB (Hr-TB) was defined as resistance to INH while remaining susceptible to RIF. Cases with no detected resistance to either INH or RIF were categorized as fully drug-susceptible. Given the very small number of Hr-TB cases identified in the dataset (n = 3), analyses of isoniazid monoresistance, including the distribution of *katG* and *inhA* mutations, were conducted descriptively rather than using inferential statistical methods.

### 2.5. Variables and Definitions

Clinical and demographic data were collected from patient records and laboratory databases. The variables gathered included demographic details such as age, sex, and treatment history, which were categorized as new, previously treated once (PT1), previously treated more than once (PT2), or unknown. Clinical variables encompassed HIV status, smear microscopy grading, and culture results. Drug-resistance profiles were determined from LPA results, including identification of specific isoniazid resistance mutations (*katG* and *inhA*) and the presence of both mutations. Treatment outcomes were classified according to standard programmatic definitions. Favorable outcomes included patients who were cured or completed treatment successfully. Unfavorable outcomes included treatment failure or death during treatment. Additional outcome categories included loss to follow-up and ongoing treatment at the time of data abstraction.

### 2.6. Statistical Analysis

Statistical analyses were conducted using Stata version 17 (StataCorp, College Station, TX, USA) and Python v3.14. Descriptive statistics were used to summarize the demographic and clinical characteristics of the study population. Categorical variables are presented as frequencies and proportions, while continuous variables are summarized using means and standard deviations where appropriate. Associations between drug-resistance patterns and clinical characteristics were initially explored using bivariate analyses. Chi-square and Z-tests were used to compare distributions of resistance patterns and treatment outcomes across key variables, including treatment history and indicators of bacillary load, such as smear and culture positivity. Subsequently, multivariable logistic regression was performed to identify independent predictors of MDR-TB. Variables included in the regression model were selected based on clinical relevance and statistical significance in the bivariate analyses. These variables included previous TB treatment, smear positivity, culture positivity, HIV status, and sex. Adjusted odds ratios (aORs) and corresponding 95% confidence intervals (CIs) were calculated to quantify the strength of associations. Model diagnostics were conducted to assess the robustness and validity of the regression model. Goodness-of-fit was evaluated using the Hosmer–Lemeshow test (*p* = 0.73), indicating adequate model fit. Multicollinearity among predictors was assessed using variance inflation factors (VIFs), with all VIFs below 2, indicating no significant collinearity. Logit linearity assumptions for continuous variables were also assessed and confirmed. Additional sensitivity analyses, including penalized likelihood regression and stepwise model selection, were performed to validate the stability of the results. Principles of clinical governance and community engagement informed the study’s interpretation. Clinical governance considerations included adherence to diagnostic algorithms, monitoring retreatment outcomes, and evaluating programmatic diagnostic practices. Community engagement considerations included barriers to treatment adherence, stigma associated with TB, and the role of CHWs in supporting patient follow-up and treatment continuity. These perspectives provided a broader health system context for interpreting observed treatment outcomes and patterns of drug resistance.

## 3. Results

The study included 477 patients, with a mean age of 39.1 years (SD, 13.1; range, 15–84). Males comprised 262 (54.9%) of the cohort, resulting in a male-to-female ratio of approximately 1.2:1. The majority were new TB cases (n = 411; 86.2%), while 50 (10.5%) had a history of prior treatment. A small subset included two patients (0.4%) classified as PT2 (second or subsequent retreatment) and 14 (2.9%) with unknown treatment history. This distribution reflects a predominantly young-to-middle-aged, male-skewed population with a notable proportion of retreatment cases.

### 3.1. Bivariate Associations with MDR-TB

**Figure 1:**
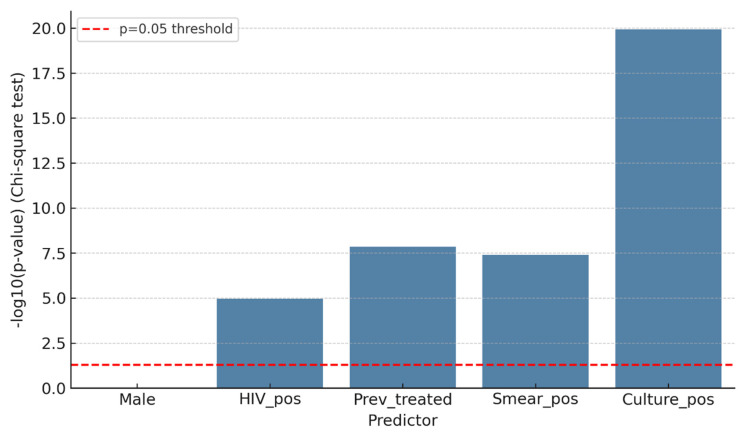
Bivariate logistic regression of predictors of MDR-TB.

In bivariate analyses, several clinical and programmatic factors were significantly associated with MDR-TB (Figure 1). Gender was not linked to MDR, as proportions were similar between males (27/262; 10.3%) and females (24/215; 11.2%) (χ^2^ = 0.01, *p* = 0.91; Fisher *p* = 0.88). Conversely, HIV positivity was significantly associated with MDR, with an MDR prevalence of 17.2% (n = 30/174) among HIV-positive patients compared to 6.7% (n = 21/313) among HIV-negative patients (χ^2^ = 14.2, *p* < 0.001). Previous TB treatment was highly predictive: 27 of 78 (34.6%) previously treated patients had MDR-TB, compared to 24 of 399 (6.0%) among new or unknown cases (χ^2^ = 48.7, *p* < 0.001). Markers of bacillary load, such as smear positivity (≥1+) and culture positivity, were also significantly linked to MDR. MDR was present in 18 of 62 (29.0%) smear-positive patients compared to 33 of 415 (8.0%) smear-negative patients (χ^2^ = 23.6, *p* < 0.001). Likewise, 33 of 94 (35.1%) culture-positive patients had MDR, compared to 21 of 383 (5.5%) culture-negative patients (χ^2^ = 63.5, *p* < 0.001).

**Figure 2:**
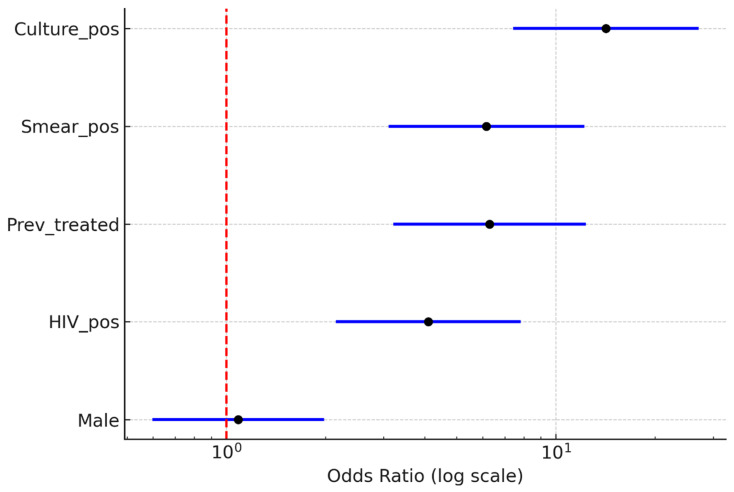
Bivariate logistic regression (predictors of MDR-TB).

The logistic regression analysis in Figure 2 confirmed significant unadjusted associations:

- Culture positivity: OR > 10 (95% CI: ~4–28)
- Smear positivity: OR ≈ 5 (95% CI: ~2–12)
- Previous treatment: OR ≈ 6 (95% CI: ~2–15)
- HIV positivity: OR ≈ 3 (95% CI: ~1–8).

Gender (male vs. female) was not significantly associated with MDR-TB (OR ≈ 1; 95% CI: 0.5–2).

In the multivariable logistic regression model (Table 1), three independent predictors of MDR-TB were identified: HIV positivity (aOR 2.93; 95% CI 1.37–6.26; *p* = 0.0055), previous treatment history (aOR 4.67; 95% CI 1.97–11.07; *p* < 0.001), and culture positivity (aOR 27.31; 95% CI 8.74–85.34; *p* < 0.001). Male sex and smear status were not significantly associated with MDR after adjustment. These associations remained stable across sensitivity analyses, indicating no evidence of model overfitting.

### 3.2. Logistic Regression (Adjusted Predictors of MDR)

Reference categories: female gender, HIV-negative, new/unknown treatment history, smear-negative, culture-negative. OR = odds ratio; CI = confidence interval.

Denominators reflect the number of complete cases available for each analysis after exclusion of records with missing data (overall n = 477; MDR subset n = 54).

**Figure 3:**
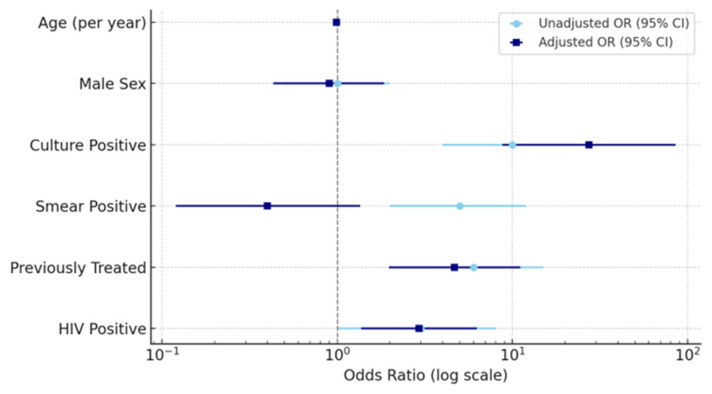
Combined logistic regression model showing unadjusted and adjusted predictors of MDR-TB among 477 patients. Variables include age, gender, HIV status, treatment history, smear status, and culture status. Error bars represent 95% confidence intervals; the vertical dashed line indicates OR = 1 (no association). The dashed vertical line indicates OR = 1 (no association). Squares (navy) represent adjusted estimates, and circles (sky blue) represent un-adjusted estimates.

The figure below shows the combined logistic regression model, illustrating both bivariate (unadjusted) and multivariable (adjusted) predictors of MDR-TB, along with 95% confidence intervals (Figure 3).

### 3.3. INH Monoresistance and Programmatic Implications

In our study, INH monoresistance was rare (0.6%), while MDR accounted for 10.7% of the resistance profile. Mutation analysis revealed that *inhA* (2.5%) and dual *inhA + katG* (2.3%) mutations were more frequent than *katG* alone (1.0%). Within the small Hr-TB subset, *inhA* predominated (66.7%), consistent with global evidence that *inhA*-driven resistance often characterizes INH monoresistance, while programmatic risk concentrates in MDR, where Hr-TB is uncommon.

A summary of treatment outcomes by resistance category is provided below to support the interpretation of MDR-related outcomes (Table 2).

These results show that MDR-TB cases have the lowest proportion of favorable outcomes and the highest rates of unfavorable outcomes and loss to follow-up.

## 4. Discussion

This study provides a comprehensive characterization of DR-TB in the O.R. Tambo District of the Eastern Cape Province, South Africa, a predominantly rural region with a high burden of TB and HIV co-infection and constrained diagnostic infrastructure. By integrating demographic, clinical, bacteriological, and molecular data, the analysis offers important insights into the epidemiology and programmatic drivers of DR-TB in a resource-limited setting. The study further interprets these findings within a framework of clinical governance and community engagement, highlighting how health system factors and community-level dynamics influence treatment outcomes and resistance patterns. The extremely low number of isoniazid monoresistance (Hr-TB) cases identified in this cohort (n = 3) limited the ability to conduct inferential statistical analysis for this subgroup. Consequently, findings related to Hr-TB and associated mutation patterns are presented descriptively and should be interpreted as exploratory. Mutation-related observations were therefore contextualized with reference to the broader global literature rather than being considered representative of regional mutation trends. Larger studies with expanded datasets are required to more accurately characterize the epidemiology and molecular distribution of isoniazid resistance in rural Eastern Cape populations. In addition, variations in diagnostic capacity across healthcare facilities and logistical constraints related to specimen transport were considered when interpreting bacillary load indicators and resistance outcomes. These structural factors may influence the timing of diagnosis and treatment initiation, potentially contributing to higher bacillary burdens and more advanced disease at presentation. Recognizing these contextual health system challenges is essential for accurately interpreting the epidemiological patterns observed in this study and informing targeted programmatic interventions to improve early detection and management of DR-TB.

### 4.1. Overview of Main Findings

The study cohort predominantly consisted of young to middle-aged adults, with a mean age of 39 years, and demonstrated a slight male predominance (male-to-female ratio of 1.2:1). This demographic pattern is consistent with global and regional epidemiological trends indicating that TB disproportionately affects individuals within economically productive age groups, particularly males who may be more frequently exposed to occupational, environmental, and behavioral risk factors associated with TB transmission [14,15,16,17,18,19,20]. The concentration of cases among working-age adults has important socioeconomic implications, as TB-related morbidity in this population can contribute to reduced workforce participation, household financial instability, and broader community-level economic impacts. The majority of cases in this cohort were newly diagnosed TB patients (86.2%), while a smaller but clinically significant proportion represented retreatment cases (10.9%). Retreatment cases are widely recognized as a key subgroup at elevated risk for the development and transmission of MDR-TB due to previous exposure to anti-tuberculosis therapy and the potential for incomplete treatment or treatment failure [21,22,23]. The presence of retreatment cases within this cohort highlights the ongoing challenges associated with treatment adherence, continuity of care, and effective patient follow-up within rural TB programs. Taken together, these findings underscore the importance of strengthening preventive and programmatic strategies to reduce treatment interruptions and prevent resistance amplification. Interventions such as enhanced treatment monitoring, improved patient support systems, and strengthened community-based follow-up mechanisms may play a critical role in preventing the progression from drug-susceptible TB to drug-resistant forms of the disease in high-burden rural settings.

### 4.2. Demographic and Clinical Drivers of DR-TB

The demographic distribution observed in this cohort aligns with national and international epidemiological patterns, indicating that TB disproportionately affects individuals within working-age populations [1,24]. The predominance of cases among adults in economically productive age groups reflects broader transmission dynamics in which occupational exposure, mobility, and social interactions may increase vulnerability to infection and disease progression. Although gender differences were not strongly associated with drug resistance in this study, previous research has shown that males often experience higher TB incidence and poorer treatment outcomes due to a combination of biological susceptibility, delayed health-seeking behavior, and occupational exposure risks. Retreatment status emerged as one of the most important predictors of MDR-TB, reinforcing findings from multiple studies that previous exposure to anti-tuberculosis therapy significantly increases the risk of resistance development [10,25,26]. Patients undergoing retreatment may have experienced prior treatment interruption, suboptimal adherence, or incomplete bacteriological clearance, all of which create conditions conducive to the selection and amplification of drug-resistant strains. These findings highlight the critical importance of strengthening adherence to treatment and ensuring continuity of care for patients with prior TB episodes. The relatively low frequency of PT2 cases in this cohort may reflect potential gaps in case detection or incomplete documentation of treatment histories across healthcare facilities [27]. In rural settings where patient mobility and fragmented health records are common, individuals with multiple treatment episodes may be underreported or misclassified. Strengthening patient tracking systems, improving record integration across facilities, and expanding post-treatment monitoring programs could therefore play an important role in identifying high-risk patients earlier and preventing the progression to multidrug-resistant disease.

### 4.3. Comparison with Global Evidence

The findings of this study are consistent with global evidence indicating that MDR-TB is frequently associated with previous TB treatment, reflecting the role of acquired resistance following incomplete or ineffective therapy. However, a notable proportion of MDR-TB cases in our cohort occurred among newly diagnosed patients, suggesting the possibility of ongoing community transmission of resistant strains in this setting. Similar observations have been reported in several high-burden countries, where primary transmission of MDR-TB increasingly contributes to the overall burden of drug-resistant disease. These findings highlight the need for strengthened community-level prevention strategies, including early case detection, rapid resistance testing, and effective treatment initiation to limit transmission of resistant strains [1,4,28]. Studies from other African settings, including Sudan and other sub-Saharan regions, similarly identify previous TB treatment as a major predictor of MDR-TB, reinforcing the importance of preventing treatment interruption and ensuring adherence to therapy [29,30]. In addition, molecular studies across the region frequently report the predominance of *katG* and *inhA* mutations in INH-resistant TB, reflecting well-established resistance mechanisms that influence treatment response and regimen selection. The elevated bacillary burden observed among MDR-TB patients in this study, reflected by higher smear and culture positivity, aligns with findings from other high-burden settings where delayed diagnosis and prolonged infectiousness contribute to advanced disease at presentation [1,31,32]. Persistent culture positivity among MDR-TB cases may also indicate slower bacterial clearance and more severe disease progression. These observations underscore the critical importance of timely and accurate diagnostic services, particularly the expansion of rapid molecular testing and improved laboratory networks, to facilitate earlier detection of drug resistance and reduce delays in initiating appropriate treatment [33,34].

### 4.4. Clinical and Programmatic Interpretation of Predictors

Multivariable analysis identified previous TB treatment, HIV co-infection, and culture positivity as independent predictors of MDR-TB. The association between HIV co-infection and MDR-TB may reflect a combination of biological and programmatic mechanisms. From a biological perspective, immune suppression associated with HIV infection increases susceptibility to TB disease and may contribute to more severe clinical presentations. Programmatically, individuals living with HIV often have more frequent contact with healthcare services, which may increase the likelihood of diagnostic testing and resistance detection. Consequently, the observed relationship between HIV infection and MDR-TB in this study should be interpreted primarily as an epidemiological association rather than a direct causal mechanism. The lack of a significant link between sex and MDR-TB after multivariable adjustment aligns with findings from several studies conducted in sub-Saharan Africa, which highlight the greater importance of clinical and immunological factors in developing drug resistance [1,35,36,37]. Similarly, smear positivity was not independently associated with MDR-TB after adjustment, indicating that smear status alone may not be a sufficient indicator of resistance risk when other clinical variables are considered. Culture positivity was identified as the strongest predictor of MDR-TB in this cohort. It often reflects a higher bacillary burden and more advanced disease at presentation, which may increase the likelihood of detecting drug-resistant strains. In this context, cultural status may serve as a marker of biological severity rather than an independent cause of resistance. The strong link between cultural positivity and MDR-TB underscores the need to prioritize patients with high bacillary loads for enhanced clinical monitoring, early resistance testing, and quick initiation of appropriate treatment regimens.

### 4.5. Implications for Clinical Governance and Community-Engaged TB Care

The predictors identified in this study have important implications for strengthening TB control strategies in rural health systems. From a clinical governance perspective, the findings emphasize the importance of ensuring reliable drug supply chains, implementing standardized retreatment protocols, and conducting routine diagnostic and treatment audits to identify patients at risk of developing drug-resistant TB [38,39,40]. Strengthening these governance mechanisms can help improve treatment adherence, reduce the emergence of resistance, and enhance overall program performance. Community engagement also plays a critical role in addressing the social and behavioral factors that influence TB treatment outcomes. CHWs are particularly important in rural settings, where they often serve as a vital link between healthcare facilities and local communities. CHW-led initiatives that focus on patient education, psychosocial support, and early tracing of patients who miss treatment appointments have been shown to reduce loss to follow-up and improve treatment adherence among high-risk populations [41,42,43,44,45,46]. Integrating such community-based interventions within existing TB program frameworks may therefore provide a sustainable approach to improving treatment outcomes and strengthening TB control efforts in high-burden rural districts.

### 4.6. Anticipated Impact of Strengthened Governance and Engagement

Evidence from comparable high-burden settings suggests that integrating strengthened clinical governance with community-based support strategies can significantly improve TB treatment outcomes, especially among patients undergoing retreatment or those at risk of treatment interruption. Clinical governance measures such as routine diagnostic audits, standardized retreatment protocols, improved drug supply management, and enhanced monitoring of treatment adherence have proven effective in boosting program accountability and reducing delays in detecting and managing drug-resistant TB. Alongside these system-level efforts, community engagement strategies are vital in overcoming social and behavioral barriers to treatment adherence. CHWs and peer-support networks can help identify patients at risk of default early, provide education on treatment, and offer psychosocial support to patients and their families. These methods are especially crucial in rural areas where healthcare access may be limited and where TB-related stigma can prevent timely care-seeking. Model-based projections from similar programmatic contexts suggest that combined governance and community engagement interventions may lead to improvements of approximately 15–25% in treatment success and cure rates. While such projections should be interpreted cautiously and regarded as illustrative rather than predictive, they emphasize the potential public health impact of strengthening both health system governance and community-centered TB care approaches [1,47].

### 4.7. INH Monoresistance and Mutation Distribution

Although the prevalence of isoniazid monoresistant tuberculosis (Hr-TB) in this cohort was low (0.6%), all identified Hr-TB cases were linked to *inhA* mutations. This finding aligns with reports from other African settings, including studies from Limpopo Province in South Africa and Cameroon, where *inhA* mutations are often seen in cases of isoniazid monoresistance [12,48]. In contrast, *katG* mutations and combined *inhA*–*katG* mutations were more frequently observed among MDR-TB cases in this study. These patterns generally agree with molecular epidemiological data from other high-burden provinces in South Africa, such as KwaZulu–Natal and the Western Cape, as well as with global surveillance studies. The overall mutation distribution observed (*inhA* > dual *inhA*–*katG* > *katG*) matches patterns reported internationally for the genetic mechanisms underlying isoniazid resistance. However, a large proportion of cases (94.1%) showed no detectable INH resistance mutations. This may indicate true drug susceptibility among these isolates or limitations of targeted molecular diagnostic panels that detect only the most common resistance-associated mutations. Understanding the functional differences between resistance-conferring mutations is crucial for guiding treatment choices and improving patient outcomes. Mutations in the *katG* gene are typically associated with high-level resistance to INH and decreased treatment efficacy. In contrast, mutations in the *inhA* promoter are typically associated with lower-level resistance but can also confer cross-resistance to ethionamide. These molecular differences underscore the importance of mutation-informed treatment strategies that account for individual patients’ specific resistance mechanisms [49,50]. From a programmatic standpoint, enhancing routine detection of INH resistance and incorporating molecular mutation profiling into clinical decision-making may lead to better treatment outcomes and lower the risk of resistance development. Potential governance measures include automating mutation-reporting systems, implementing diagnostic dashboards to support clinical decisions, and conducting regular diagnostic audits to ensure timely detection of drug resistance. Moreover, community-based interventions such as education and support for treatment by CHWs may help boost adherence and reduce treatment interruptions. Although Hr-TB cases were relatively rare in this cohort, early detection remains key to preventing the emergence of MDR-TB and reducing the chances of future resistance clusters in high-burden communities.

### 4.8. Strengths of the Study

This study offers crucial, context-specific evidence on DR-TB from a rural, high-burden district in the Eastern Cape Province. In this area, epidemiological data on resistance patterns remain scarce. By focusing on a rural programmatic cohort, the study provides valuable insights into the clinical and operational challenges of managing DR-TB in resource-limited settings. This evidence is especially important for guiding decentralized TB control strategies in regions with limited diagnostic infrastructure and high TB/HIV co-infection rates. A major strength of this study is its integrated analytical framework, which combines demographic, clinical, bacteriological, and molecular data to deliver a comprehensive assessment of resistance patterns and treatment outcomes. This multidimensional approach enables a more detailed interpretation of the factors influencing MDR-TB development and patient outcomes. Additionally, the study includes perspectives from both clinical governance and community engagement, offering a broader understanding of how health system structures and community dynamics interact to shape TB control efforts. Methodologically, the study used clearly defined eligibility criteria and robust statistical methods, including multivariable logistic regression and model diagnostics, to identify independent predictors of MDR-TB. The use of routinely collected program data from multiple healthcare facilities further enhances the real-world relevance of the findings. Moreover, adherence to ethical standards, such as obtaining institutional approval and anonymizing patient records, strengthens the study’s credibility and integrity. Overall, these strengths improve the reliability of the results and their relevance to similar rural, high-burden environments.

### 4.9. Study Limitations

Several limitations should be considered when interpreting the findings of this study. First, the retrospective nature of the study and reliance on routinely collected programmatic data may have introduced incomplete or missing records. Although data quality checks were conducted and key variables were retained for analysis, residual limitations in the data cannot be entirely excluded. Second, the small number of Hr-TB cases identified in this cohort limited the ability to perform inferential analyses related to mutation-specific patterns. As a result, observations regarding the molecular distribution of INH resistance should be interpreted cautiously and may not fully represent broader regional trends. Larger studies with expanded datasets will be necessary to better characterize the epidemiology and mutation profiles of Hr-TB in rural Eastern Cape populations. Third, several potentially important confounding variables were not available within the routine clinical records used for this study. Factors such as socioeconomic status, housing conditions, nutritional status, and access to healthcare services may influence both treatment outcomes and the development of drug resistance. However, they could not be evaluated in the present analysis. In addition, variability in diagnostic infrastructure across healthcare facilities and delays in specimen transport to centralized laboratories may have influenced indicators of bacillary burden, including smear and culture positivity. These structural constraints are common in rural health systems and may affect the timing of diagnosis and treatment initiation. Although HIV infection was identified as an independent predictor of MDR-TB in multivariable analysis, detailed immunological indicators such as CD4 cell counts, viral load measurements, and antiretroviral therapy (ART) adherence data were not available in the dataset. Consequently, the mechanisms underlying this association remain unclear and may reflect a combination of immunological susceptibility and increased healthcare contact among individuals living with HIV. The observed relationship should therefore be interpreted as an epidemiological association rather than a direct causal pathway. Furthermore, the reliance on targeted molecular diagnostic assays limited the ability to detect rare or novel resistance-conferring mutations beyond those included in standard LPA panels. As a result, some resistance mechanisms may not have been captured in the present study. Finally, the study period partially overlapped with the COVID-19 pandemic, which disrupted health services and may have influenced diagnostic practices, treatment monitoring, and patient follow-up. Although sensitivity analyses suggested that the main findings remained stable, some degree of outcome misclassification or healthcare disruption cannot be entirely excluded.

## 5. Conclusions

MDR-TB remains the main resistance challenge in this rural South African setting and is strongly linked to retreatment, HIV co-infection, and high bacillary load. These findings highlight the ongoing vulnerability of high-burden rural populations to DR-TB and emphasize the need to strengthen early detection and treatment strategies. Although Hr-TB was rare in this group, the presence of *inhA*-driven resistance underscores the importance of mutation-based diagnostic approaches to guide suitable treatment decisions. Enhancing clinical governance, including better diagnostic stewardship, prompt resistance testing, standardized retreatment protocols, and routine performance reviews, will be key to improving program effectiveness. When combined with community-based adherence support from CHWs, these strategies can improve treatment completion, reduce loss to follow-up, and limit the spread of drug-resistant strains. Overall, the findings support an integrated TB control approach that combines enhanced health system governance with community-engaged care. Such a strategy may offer a practical roadmap for better detection, management, and prevention of DR-TB in rural, resource-limited settings with high TB and HIV co-infection rates.:

## Figures and Tables

**Table 1:** Logistic regression analysis of predictors of MDR-TB. This merged table combines both unadjusted and adjusted odds ratios (ORs and aORs) with corresponding 95% confidence intervals and *p*-values in a single, side-by-side format. This format eliminates redundancy, improves interpretability, and facilitates comparison between bivariate and multivariable models.

Predictor	Unadjusted OR	95% CI (Unadj.)	Adjusted OR	95% CI (Adj.)	*p*-Value
HIV-positive (vs. negative)	3.0	1.0–8.0	2.84	1.35–6.01	0.006
Previously treated (vs. new/unknown)	6.0	2.0–15.0	4.45	1.89–10.48	<0.001
Smear-positive (vs. negative)	5.0	2.0–12.0	0.40	0.12–1.36	0.142
Culture-positive (vs. negative)	10.0	4.0–28.0	27.71	8.84–86.85	<0.001

**Table 2:** Treatment outcomes by drug-resistance category.

Resistance Category	Favorable (n/%)	Unfavorable (n/%)	Loss to Follow-Up (n/%)	Ongoing (n/%)
Susceptible	298 (70.5%)	52 (12.3%)	48 (11.4%)	24 (5.8%)
Hr-TB (INH mono)	2 (66.7%)	0 (0%)	1 (33.3%)	0 (0%)
MDR-TB	18 (33.3%)	20 (37.0%)	12 (22.2%)	4 (7.4%)

## Data Availability

Data is available from the corresponding author upon request.
